# Omega-3 Fatty Acids for Depression in Multiple Sclerosis: A Randomized Pilot Study

**DOI:** 10.1371/journal.pone.0147195

**Published:** 2016-01-22

**Authors:** Lynne Shinto, Gail Marracci, David C. Mohr, Lauren Bumgarner, Charles Murchison, Angela Senders, Dennis Bourdette

**Affiliations:** 1 Department of Neurology, Oregon Health & Science University, Portland, OR, United States of America; 2 Research Service and VA Multiple Sclerosis Center of Excellence-West, Portland, OR, United States of America; 3 Department of Preventive Medicine, Northwestern University, Chicago, IL, United States of America; National Cancer Center, JAPAN

## Abstract

Multiple sclerosis is the most common chronic disabling disease in the central nervous system in young to middle aged adults. Depression is common in multiple sclerosis (MS) affecting between 50–60% of patients. Pilot studies in unipolar depression report an improvement in depression when omega-3 fatty acids are given with antidepressants. The objective of this study was to investigate whether omega-3 fatty acid supplementation, as an augmentation therapy, improves treatment-resistant major depressive disorder (MDD) in people with MS. We performed a randomized, double-blind, placebo-controlled pilot study of omega-3 fatty acids at six grams per day over three months. The primary outcome was a 50% or greater improvement on the Montgomery-Asberg Depression Rating Scale (MADRS). Thirty-nine participants were randomized and thirty-one completed the 3-month intervention. Improvement on MADRS between groups was not significantly different at the 3-month end point with 47.4% in the omega-3 fatty acid group and 45.5% in the placebo group showing 50% or greater improvement (p = 0.30). Omega-3 fatty acids as an augmentation therapy for treatment-resistant depression in MS was not significantly different than placebo in this pilot trial. Omega-3 fatty acid supplementation at the dose given was well-tolerated over 3 months.

**Trial Registration:**

ClinicalTrials.gov NCT00122954

## Introduction

Multiple sclerosis (MS) is the most common chronic disabling disease of the central nervous system affecting young and middle aged adults with a typical onset age between 20 and 55 years. Women are more commonly affected than men with about 60% of cases being female and MS is most common among white/Caucasians, particularly those of Northern European descent [1]. It is estimated that 50–60% of patients with MS are affected with depression at some point in their lives [2].

In the general population, although antidepressant medication show benefit in alleviating depression, 29%-46% of depressed patients show a partial to no response to antidepressant medication therapy [3]. The treatment strategy for these patients includes any one of the following, increasing medication dose, switching to a different type of antidepressant medication, or augmenting current medication with an additional antidepressant [4]. A large, multi-center study that evaluated different treatment strategies for people with treatment-resistant major depressive disorder (MDD) found that 20–30% of participants achieved a remission in depression by “switching” or “augmenting” their antidepressant medication. This study also found that 21–27% of those randomized to the “switching” group and up to 20% of the “augmentation” group did not achieve remission from depression and had to discontinue the study because of intolerable side effects [5–6]. Although remission can be achieved in a subset of MDD patients using these strategies, there are a considerable number of patients that do not tolerate “switching” or “augmenting” antidepressant medication. Identifying safe and effective therapies for treatment-resistant MDD in MS is warranted given the high prevalence of depression and the high use of antidepressant medication in people with MS.

Omega-3 fatty acids (omega-3 FA) have been reported to improve depression with low adverse effects when used as an augmentation therapy in unipolar depression [7–10]. The primary aim of this pilot study was to determine if omega-3 FA supplementation is an effective and safe augmentation therapy for treatment-resistant MDD in MS. The secondary aim was to evaluate omega-3 FA effects on quality of life.

## Materials and Methods

### Ethics statement

This study was approved and monitored by Oregon Health & Science University’s (OHSU) Institutional Review Board on June 20^th^, 2005 and registered at ClinicalTrials.gov as NCT00122954 on July 20^th^, 2005. All participants were informed about the study procedures and potential risks and gave written consent before entering the study.

### Participants

Inclusion criteria were age 18–85 years, diagnosed with MS by McDonald criteria [11], all types of MS (relapsing remitting and progressive types), major depressive disorder (MDD) by Diagnostic and Statistical Manual of Mental Disorder-IV criteria (DSM-IV) based on Structured Clinical Interview for DSM (SCID) for Axis I disorders [12], mild to moderate depression (BDI-I score 10–30) [13–14], stable dose of antidepressant medication three months prior to study enrollment, and if on a MS disease modifying medication, on stable dose for 6 months prior to study enrollment. Exclusion criteria were, severe depression with BDI > 30 and MADRS > 30 [13–15], MS exacerbation or corticosteroid therapy within one month prior of enrollment, Mini-Mental State Examination (MMSE) ≤ 24, pregnancy, current or past history of significant ventricular arrhythmia, and general health status that would interfere with study participation. Potential confounds with naturally occurring sources of omega-3 fatty acids were addressed by excluding participants who reported fish oil or cod liver oil supplementation within 30 days of enrollment, and greater than one 6-ounce serving per week of fish or seafood within 30 days of enrollment.

### Study design and randomization

This study was designed as a parallel group, randomized, double-blind placebo-controlled pilot clinical trial to test the safety and efficacy of omega-3 fatty acids as an augmentation therapy to antidepressants over 3 months. Fifty percent improvement in MADRS scores after 3 months of treatment was used as the primary outcome measure as this would be considered a clinically significant change in depression [14–15]. The study used reported data from Nemets et al. for sample size and power calculations [7]. Nemets et al. evaluated 2g/day of ethyl-EPA as an adjunct therapy in a double blind placebo controlled trial in 20 subjects with unipolar depression and showed a mean 9.3-unit difference in HDRS between placebo and treatment groups over 4-weeks (p<0.001); using this data a sample size of 11 subjects per group provides 80% power with an alpha = 0.05, to detect the above difference in HDRS between the treatment and placebo group. The study was powered to enroll twenty six subjects (13/group), the number needed to see a difference in Hamilton Depression Rating Scale (HDRS) over 3 months that factors in a 20% drop out. Our study used the MADRS instead of HDRS for two reasons, 1) the MADRS has good validation with the HDRS (15), and 2) the MADRS is more sensitive to emotional change having less weight on somatic components than the HDRS [16]. After meeting eligibility criteria, participants were randomized to receive either placebo or omega-3 fatty acids by an independent pharmacist who dispensed either active or placebo capsules according to a computer generated randomization list. Participants were stratified into two categories ‘mildly depressed‘ (BDI-I score 10–17) and ‘moderately depressed‘ (BDI-I score.18-30), to insure that each group contained equal numbers of mild and moderately depressed subjects.

### Treatment

The product in this study is fish oil capsules that contain both eicosapentaenoic acid (EPA) and docosahexaenoic acid (DHA) with a EPA: DHA ratio of 1.4 to 1.0 in a triglyceride form supplied by Nordic Naturals, Inc. (Watsonville, CA). Each gel capsule contains 0.968 grams of fish oil with 0.325 grams EPA and 0.225 grams DHA and 0.640 grams of total omega-3 fatty acids. Participants randomized to the omega-3 FA group received a daily dose of 6 capsules (1.95 grams of EPA and 1.35 grams of DHA). Participants randomized to the placebo group received capsules that contained soybean oil with 1% fish oil so that it was flavored to taste and smell similar to the fish oil capsules. Fish oil concentrate and placebo capsules were supplied by Nordic Naturals, Watsonville, CA. Both groups took 3 capsules in the morning and 3 capsules in the afternoon with food or a meal.

### Depression measures

The SCID is a semi-structured interview that was administered by a trained clinician or research associate to assess major Axis I disorders [12] and was used to assess MDD at screening. The MADRS is a structured interview assessment of depression, designed to be especially sensitive to changes in a patient’s depression symptoms after antidepressant therapy and is more oriented towards psychic rather than somatic symptoms of depression [14–16]. The Department of Psychiatry at OHSU supervised the training and administration of the SCID and MADRS. Inter-rater reliability was assessed throughout the study to ensure a kappa value of ≥ 0.90. In addition to 50% or greater improvement in MADRS, treatment effect on change in mean MADRS scores over 3 months was also evaluated. The Beck Depression Inventory, version 1 (BDI-I) is a self-report measure of depression and is commonly used in MS studies because it is an easily administered depression measure [13,17]. We used the BDI-I to evaluate severity of depression for study inclusion and used improvement on the BDI-I as a secondary outcome measure. The Expanded Disability Status Scale (EDSS) is a clinician-rated scale used to quantify disability in MS and ranges from 0 (no disability) to 10 (dead) [18]. EDSS was used to evaluate the potential effects of disease severity on depression outcomes.

### Quality of life

The SF-36 is one of the most commonly used measures of health-related quality of life (HRQL) [19], treatment effect on physical and mental component summary scores (PCS and MCS) was evaluated over 3 months. The SF-36 was self-administered and reviewed by study coordinators during study visit so that they were complete after each study visit.

### Omega-3 fatty acids

Omega-3 fatty acids, docosahexaenoic acid (DHA) and eicosapentaenoic acid (EPA), were measured in red blood cell (RBC) membranes in methods described by Ruyle et al. at OHSU’s Lipid Lab. RBC fatty acids are good indicators for tissue fatty acid levels [20]. EPA and DHA levels are indicated by percent of total fatty acids measured in RBC membranes, a higher percent reflects higher amounts of DHA and EPA incorporated into membranes.

### Compliance and blinding

Compliance was assessed by pill count at 3 months. The study assessed the maintenance of blinding over 3 months by asking participants and all research staff involved in administering outcome measures about knowledge of group assignment at 3 months.

### Safety

Safety was evaluated by adverse events reported by participants, laboratory tests (comprehensive metabolic panel and prothrombin time (PT/INR)), vital signs, and physical and neurological examinations.

### Statistical analysis

An intention to treat analysis was used to evaluate all outcomes. Baseline characteristics were compared between groups using Fisher’s exact tests for categorical variables and analysis of variance (ANOVA) for continuous variables. The primary outcome measure was 50% or greater improvement in MADRS scores at 3 months. Secondary outcome measures included 50% or greater improvement in BDI scores at 3 months and change in quality of life (PCS and MCS) over 3 months. For the primary outcome a mixed effects logistic regression model was used, the dependent variable was a dichotomous variable indicating whether or not 50% or greater reduction in MADRS score was achieved at 3 months, the model was adjusted for age and MS disease duration. The group difference in slope was assessed by the interaction of intervention time by omega-3 FA group using the placebo group as the reference. Improvement in BDI was assessed using the same analytic method. For quality of life (PCS, MCS) linear mixed effects model was used, this method allows for correlation between repeated observations on each subject and this model and mixed effects logistic regression model provides valid inference in the presence of missing data as long as the data is missing at random (MAR). For logistic and linear mixed effects models, each outcome was set as the dependent variable, independent variable included treatment group, age, and MS disease duration (years since first symptom), in a separate model we included age and MS disability (EDSS) and age and education level as independent variables. The group difference in slope was assessed by the interaction of intervention time by ω-3 group using the placebo group as the reference. SPSS software was used for all outcomes analysis.

## Results

### Patient characteristics

**Fig 1:**
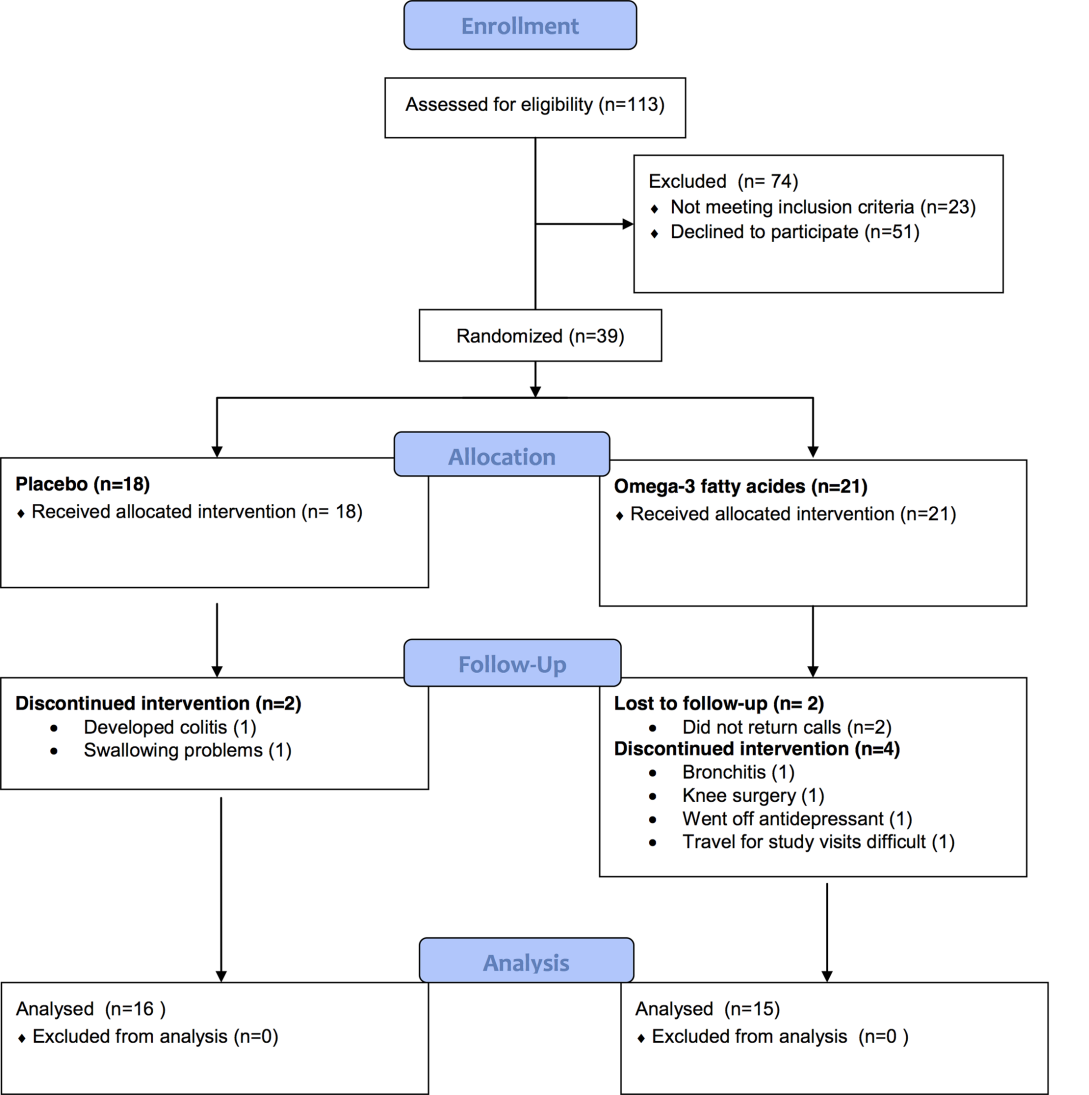
Consort Flow Chart.

The CONSORT (Consolidated Standards of Reporting Trials) flowchart for the study is presented in Fig 1. A total of 39 participants were randomized, placebo (n = 18) and omega-3 fatty acids (n = 21). There was a 20.5% drop out before the 3-month primary endpoint analysis, 2 subjects from the placebo group and 6 subjects from the omega-3 FA group (Fig 1). Per protocol all participants were on antidepressant medication, 66.7% of the placebo group and 88.9% of the omega-3 FA group were adequately dosed [21] with no significant difference between groups being adequately dosed (p = 0.23) (Table 1 and S1 Table). The only significant difference between groups in baseline characteristics was that the omega-3 FA group was more highly educated with 72.2% having a college degree or higher compared to 27.8% in the placebo group (p = 0.05) (Table 1). As expected, omega-3 FA levels were significantly increased at 3-months when compared to baseline levels in the group receiving fish oil concentrate, while the group receiving placebo oil showed no difference from baseline in EPA and DHA levels (Table 2).

**Table 1:** Baseline characteristics.

	Placebo	Omega-3 FA	P Value
	Mean (SD) or n (%)	Mean (SD) or n (%)	
Age (mean years, SD)	51.9 (10.0)	50.7 (11.6)	0.73
Female	17 (94.4)	19 (90.5)	1.00
White	18 (100)	21 (100)	1.00
College or greater	5 (27.8)	13 (72.2)	0.05
Disease duration (mean years, SD)	17.2 (10.0)	16.6 (9.5)	0.86
EDSS (mean, SD)	4.1 (2.4)	3.2 (2.1)	0.24
MADRS (mean, SD)	19.1 (4.0)	18.4 (5.3)	0.63
BDI (mean, SD)	19.6 (5.7)	20.1 (8.0)	0.83
EPA (% total fatty acids, mean, SD)	0.61 (0.3)	0.58 (0.2)	0.74
DHA (% total fatty acids, mean, SD)	3.9 (1.2)	4.0 (1.0)	0.93
Adequate antidepressant use^a^	12 (66.7)	16 (88.9)	0.23
MS DMT Use	12 (66.7)	13 (61.9)	1.00

EDSS: Expanded Disability Status Scale; MADRS: Montgomery-Asberg Depression Rating Scale; BDI: Beck Depression Inventory; EPA: Eicosapentaenoic Acid measured from red blood cell membranes; DHA: Docosahexaenoic Acid measured from red blood cell membranes; MS DMT Use: MS Disease Modifying Therapy use includes: glatiramer acetate, interferon beta-1a, interferon beta-1b.

^a^Per protocol all participants were on one antidepressant medication. Adequate antidepressant dose is defined as minimal clinical dose to two times minimal clinical dose (S1 Table).

**Table 2:** Percent of total fatty acids in red blood cell membranes.

	Baseline Mean (SEM)	3 Months Mean (SEM)	^a^P Value
DHA			
Placebo	3.9 (0.06)	4.1 (0.3)	0.3
ω-3	3.9 (0.3)	7.3 (0.3)	<0.001
EPA			
Placebo	0.6 (0.3)	0.95 (0.3)	0.66
ω-3	0.6 (0.07)	3.99 (0.42)	<0.001
			

SEM: standard error of the mean.

^a^Change from baseline to 3-months.

### Depression outcomes

**Fig 2:**
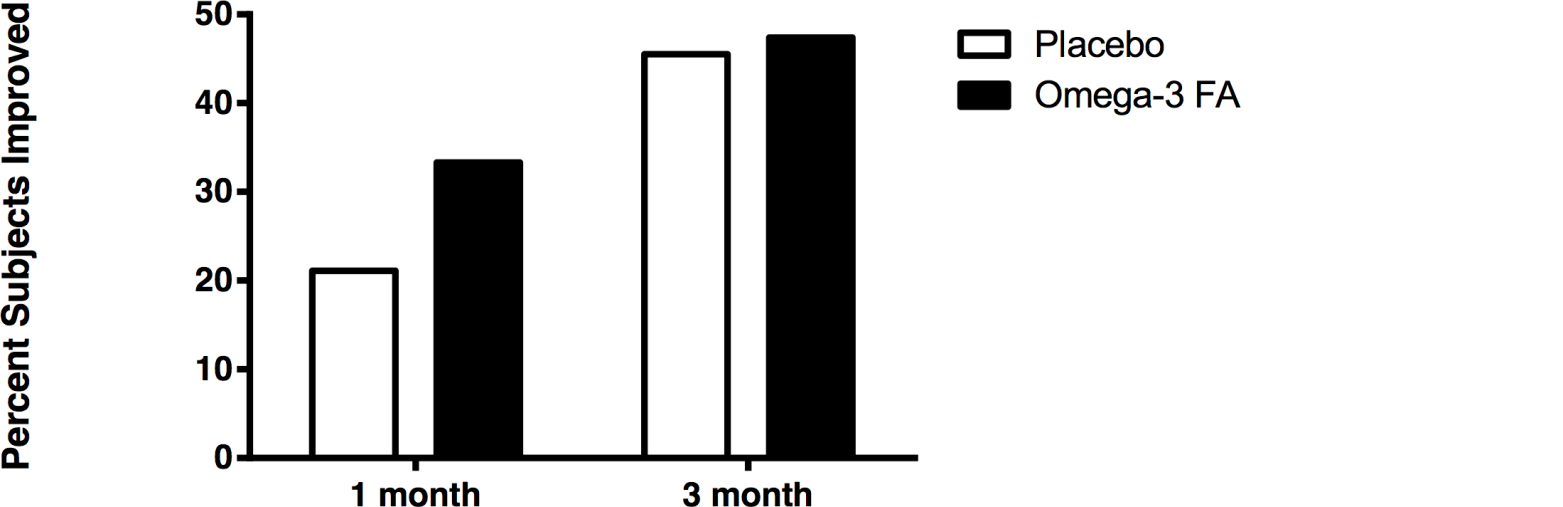
MADRS Improvement. This figure reflects percent of subjects improved by 50% or more from baseline MADRS score. Mixed effects logistic regression model adjusted for age and MS disease duration. No difference between placebo and omega-3 FA was found over 3 months (p = 0.30).

**Fig 3:**
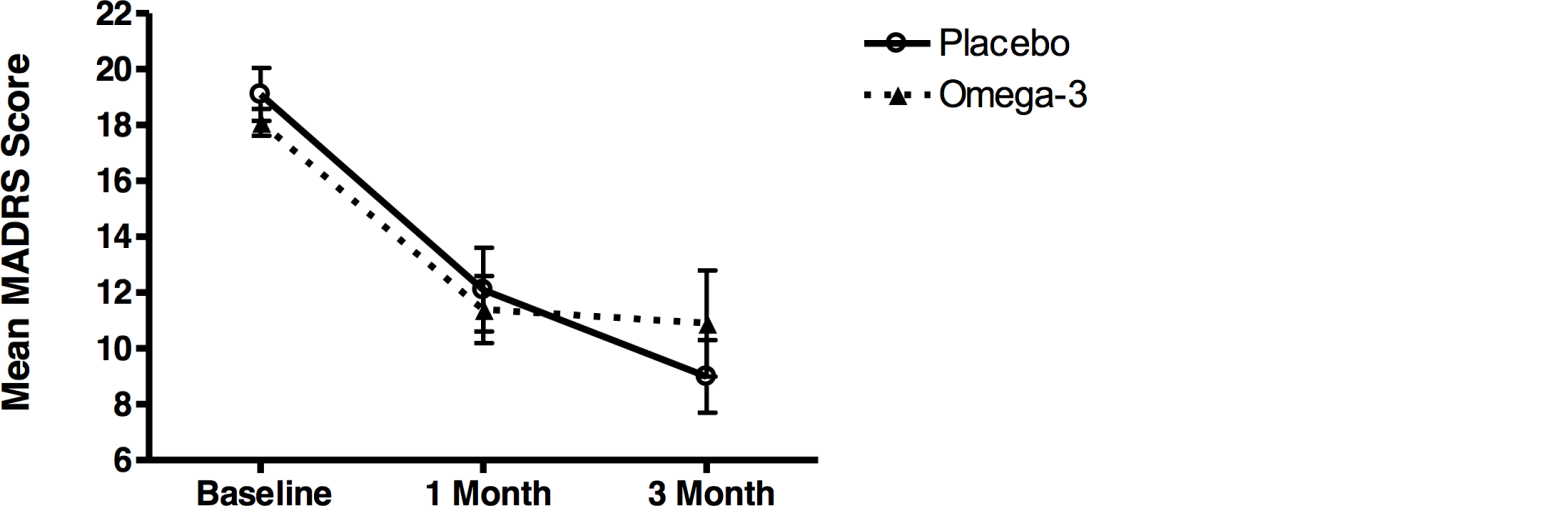
Mean MADRS. Linear mixed effects model adjusted for age and MS disease duration, error bars indicate standard error of the mean. No difference between placebo and omega-3 FA was found over 3 months (p = 0.23).

There was no difference between groups the primary outcome measure (50% or greater improvement on MADRS) at 1 and 3 months (Fig 2 and S2A Table). At 1-month 21.1% of the placebo group and 33.3% of the omega-3 FA group had ≥ 50% improvement in MADRS and at 3-months 45.5% of the placebo group and 47.4% of the omega-3 FA group had ≥ 50% improvement in MADRS. In a linear mixed effects model adjusted for age and MS disease duration there was no difference in MADRS improvement between groups from baseline to 3-months (p = 0.30). There was no difference between groups in total MADRS scores at 3-months (p = 0.23) (Fig 3, S2B Table). In a linear mixed effects model adjusting for age and disease duration there was no difference between BDI improvement at 3-months (p = 0.20), and there was no difference between groups in total BDI scores at 3-months (p = 0.27) (S1 and S2 Figs and S2C and S2D Table).

### Quality of life

There was no difference between groups in quality of life, physical component summary (PCS) (p = 0.10) and mental component summary (MCS) (p = 0.06) at the 3-month end point. The placebo group showed no significant change in PCS from baseline to end-of-treatment (mean change –0.8 SEM ± 0.8 (p = 0.29)), while the omega-3 FA group showed a trend-level positive change in PCS from baseline to end-of-treatment (mean change 1.6 SEM ± 0.8 (p = 0.06)). Both groups showed a significant positive change in MCS from baseline to 3-months, the placebo group showed a mean change of 4.2 SEM ± 1.0 points (p<0.01), while the omega-3 FA group showed a mean change of 1.5 SEM ± 1.0 (p = 0.16). All analyses were adjusted for age and disease duration.

### Safety, compliance, and blinding

Omega-3 FA in the form of fish oil concentrate capsules was well tolerated, and there were no serious adverse event (Table 3). All mean values for the comprehensive metabolic panel and PT/INR, except for glucose levels in the omega-3 group (107.8 mg/dL), were within normal limits at 3-months. Participants were not fasted before blood draws. When asked about treatment assignment at the end of the study the majority reported no knowledge of treatment assignment, research staff (100%), placebo subjects (75%), and omega-3 FA subjects (80%). Compliance by pill count was > 90% for both groups.

**Table 3:** Adverse events.

Type of AE	Placebo	Omega-3 FA	P Value
Total AE	16/18	11/21	0.26
^a^MS symptoms	8/18	1/21	0.03
Cold/Flu	3/18	3/21	1.00
Urinary Tract Infection	4/18	0/21	0.11
^b^Gastrointestinal Upset	2/18	2/21	1.00
Bruising	0/18	3/21	0.10

Total AE and Top 5 types of AE reported, comparison between groups using Fisher’s Exact Test.

^a^MS symptoms include fatigue, numbness/tingling, MS exacerbation.

^b^GI upset include acid reflux, diarrhea/loose stools, colitis, nausea.

## Discussion

This is the first study to evaluate omega-3 FA as an augmentation strategy for treatment-resistant MDD in MS. The study found no difference between groups on the primary outcome measure, 50% or greater improvement in the MADRS. In addition, we saw no difference between groups in mean total MADRS or BDI scores at 3-months. Both groups had significant improvements from baseline on all measures of depression after 3-months and were therefore indistinguishable. Four previous pilot studies that evaluated omega-3 FA as adjunct therapy in MDD without comorbid disease have reported a significant improvement in the Hamilton Depression Rating Scale (HDRS) when compared to placebo [7–10]. In common with the previous four studies we used DSM-IV criteria for MDD, evaluated a similar per group sample size and omega-3 FA dose, and used a clinician-rated depression measure [7–10]. The MADRS was chosen in this study because it is reported to be less influenced by somatic symptoms than the HDRS and therefore more sensitive to changes in emotional well-being [16]. It is not likely that using the MADRS, instead of the HDRS, significantly influenced the ability to detect a treatment effect as the MADRS is reported to have good validation with the HDRS and is a good measure of clinically significant changes in depression [15–16].

Our pilot study had a very high placebo response that obscured our ability to detect a treatment effect at 3-months. A meta-analysis conducted on the US Food and Drug Administration (FDA) database reports that approximately 50% of clinical studies evaluating antidepressant medication failed to show a statistically significant difference between drug and placebo because of a high placebo response, and that the placebo effect is estimated to account for 67% of the treatment effect in patients receiving antidepressants for unipolar depression [22]. Some factors reported to influence placebo response in depression studies include, depression severity (higher placebo response in mild to moderate depression compared to severe), use or non-use of a placebo run-in phase, and participant expectancy [23–24]. This pilot study was well-blinded, 75% or greater in both groups remained blinded to treatment, which decreases the placebo effect from participant expectancy. The study evaluated subjects with mild to moderate depression and did not implement a placebo run-in phase which may explain the high placebo response diminishing our ability to see a treatment effect. For future studies in MS depression, expectancy and its association with placebo response should be monitored as well implementing a placebo run-in phase. In studies evaluating omega-3 FA in unipolar depression, it is still unclear whether or not implementing a placebo run-in phase improves outcomes, as use of this method has yielded mixed outcomes. Two studies that implemented a placebo run-in phase to exclude responders during this phase reported a significant improvement in depression in the omega-3 FA compared to placebo [9–10]. Three studies that did not implement a placebo run-in phase: two showed a significant improvement in the omega-3 FA group [7–8] and one did no show a difference between groups [25].

There was no significant difference in quality of life (MCS or PCS) between groups. It should be noted that change from baseline to 3-months for each group was evaluated and both groups showed an improvement in MCS that corresponds with our findings in improvement in both MADRS and BDI. The study’s sample size was relatively small, although it was powered to see a difference in our primary outcome between groups with a total of 26 subjects (13 per group). At three months 31 subjects completed the study which was a sample size adequately powered to see a treatment difference.

Strengths of the study include a randomized, placebo-controlled design, use of a clinically meaningful primary outcome measure, high compliance by pill count and RBC membrane fatty acid analysis, and assessments of participant and outcome assessor blinding. Omega-3 FA was well tolerated over 3 months and in adverse events reports there is some indication that MS symptoms in the omega-3 FA were reduced compared to the placebo group (Table 3). This observation combined with improvement for the omega-3 FA group in PCS (p = 0.06) may indicate a hint of benefit of omega-3 FA in MS unrelated to depression that may warrant further exploration.

### Limitations

The majority of our participants were white/Caucasian women which limits the generalizability of our results. Baseline characteristic revealed an imbalance in education levels between groups with a higher percentage in the omega-3 FA group having a college education or higher (Table 1). In a mixed effects model analysis we adjusted for education level (college degree or higher versus lower than college degree) and age on each depression outcome (MADRS improvement, Total MADRS, BDI improvement, Total BDI) and did not see a significant effect on any outcome (p-values >0.35 on all measures). Per protocol all of the study participants were on a stable dose of an antidepressant medication but not all were adequately dosed (Table 1 and S1 Table) and this could have affected depression outcomes. When we examined adequate dosing in a mixed effects model (0 = not adequately dosed and 1 = adequately dosed) on each depression outcome, we found that having an adequate dose would increase the odds of improvement in MADRS (50% or greater) by 3.55, the model showed no difference between the placebo or omega-3 FA groups on this outcome (p = 0.54) as the majority in both groups were adequately dosed and relatively balanced in percentage of participants that were adequately dosed (66.7% of placebo, 88.9% of omega-3 FA).

Many MS symptoms can mimic MDD symptoms, these symptoms include sleep disturbance, fatigue, decreased work/activities from physical disability, making it difficult to completely separate symptoms that are solely MS from those that are solely MDD. The SCID to assess Axis I disorders for MDD was not altered to address symptoms that might reflect MS, therefore the study is limited in that some MDD may also reflect MS symptoms. Published studies that use SCID to assess Axis I disorders have not discussed alteration of this interview to address potential confounding with MS symptoms [26–27]. The MADRS was chosen as our primary outcome measure because it is reported to be less influenced by somatic symptoms than the HDRS and therefore more sensitive to changes in changes of emotional well-being in people with physical illness [16].

## Conclusions

Omega-3 FA as an augmentation therapy for treatment-resistant depression in MS is not significantly different than placebo. Omega-FA appeared to be safe and well-tolerated over 3 months. Whether implementation of a placebo run-in phase can decrease placebo effect in MS depression studies and whether omega-3 FA possess benefit in other MS-related symptoms remains to be determined.

## Supporting Information

S1 Checklist CONSORT 2010 Checklist. (DOC)

S1 Dataset Due to the presence of of identifying patient information, the underlying dataset is available upon request from the authors (shintol@ohsu.edu). (XLS)

S1 Fig BDI Improvement. Mixed effects logistic regression model adjusted for age and MS disease duration. No difference between placebo and omega-3 FA was found over 3 months (p = 0.20).
(TIFF)

S2 Fig Mean BDI. Linear mixed effects model adjusted for age and MS disease duration, error bars indicate standard error of the mean. No difference between placebo and omega-3 FA was found over 3 months (p = 0.27).
(TIFF)

S1 Protocol IRB Protocol June 06, 2005. (DOC)

S1 Table Participant Antidepressant Dose. (DOCX)

S2 Table Parameter Estimates for MADRS and BDI Mixed Model Analysis. (DOCX)
