# Supplementary figures and images for: Omega-3 Fatty Acids for Depression in Multiple Sclerosis: A Randomized Pilot Study
Source: PLoS One. 2016 Jan 22;11(1):e0147195. doi: 10.1371/journal.pone.0147195 (PMC4723316; doi:10.1371/journal.pone.0147195)

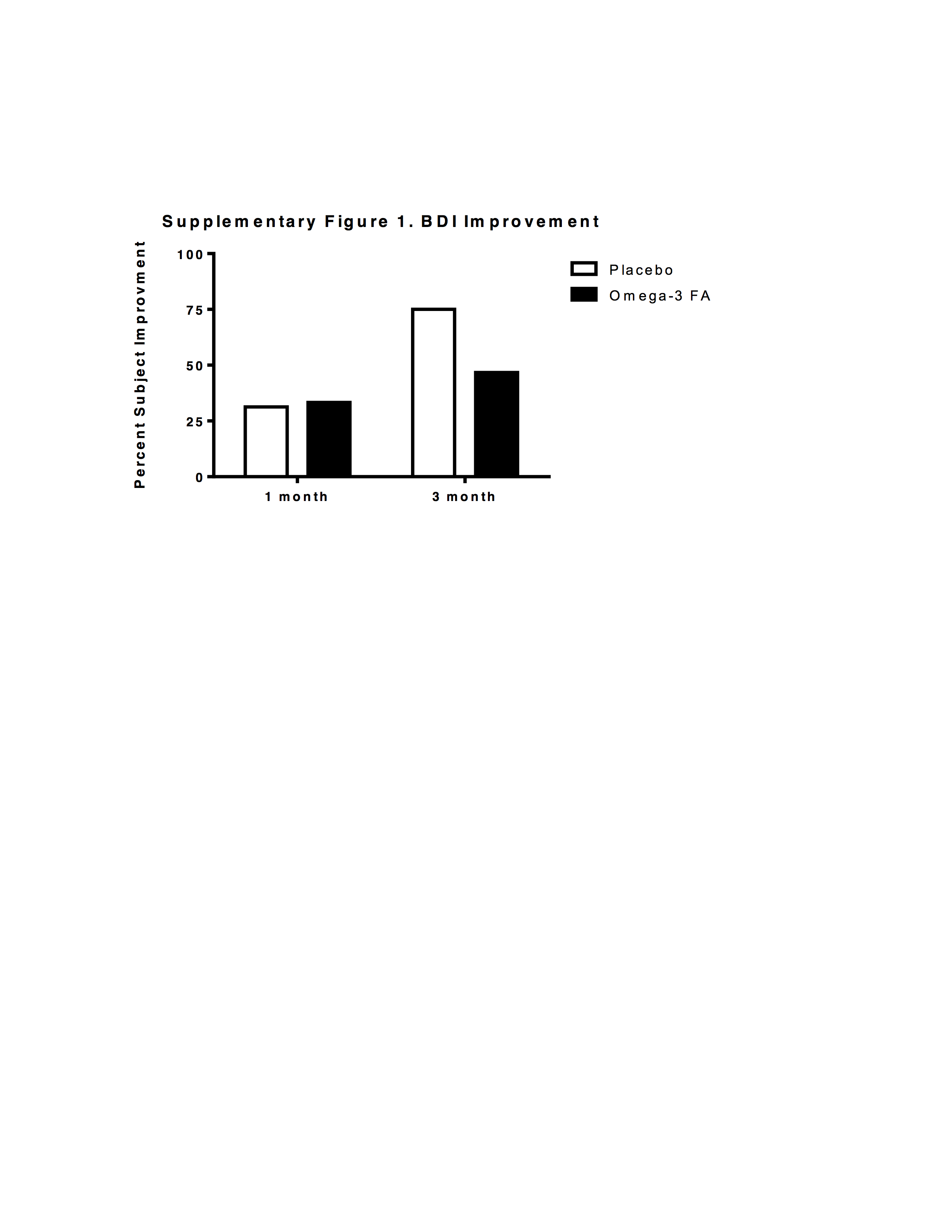

Supplement: S1 Fig — Mixed effects logistic regression model adjusted for age and MS disease duration. No difference between placebo and omega-3 FA was found over 3 months (p = 0.20). (TIFF) [file pone.0147195.s003.tiff]

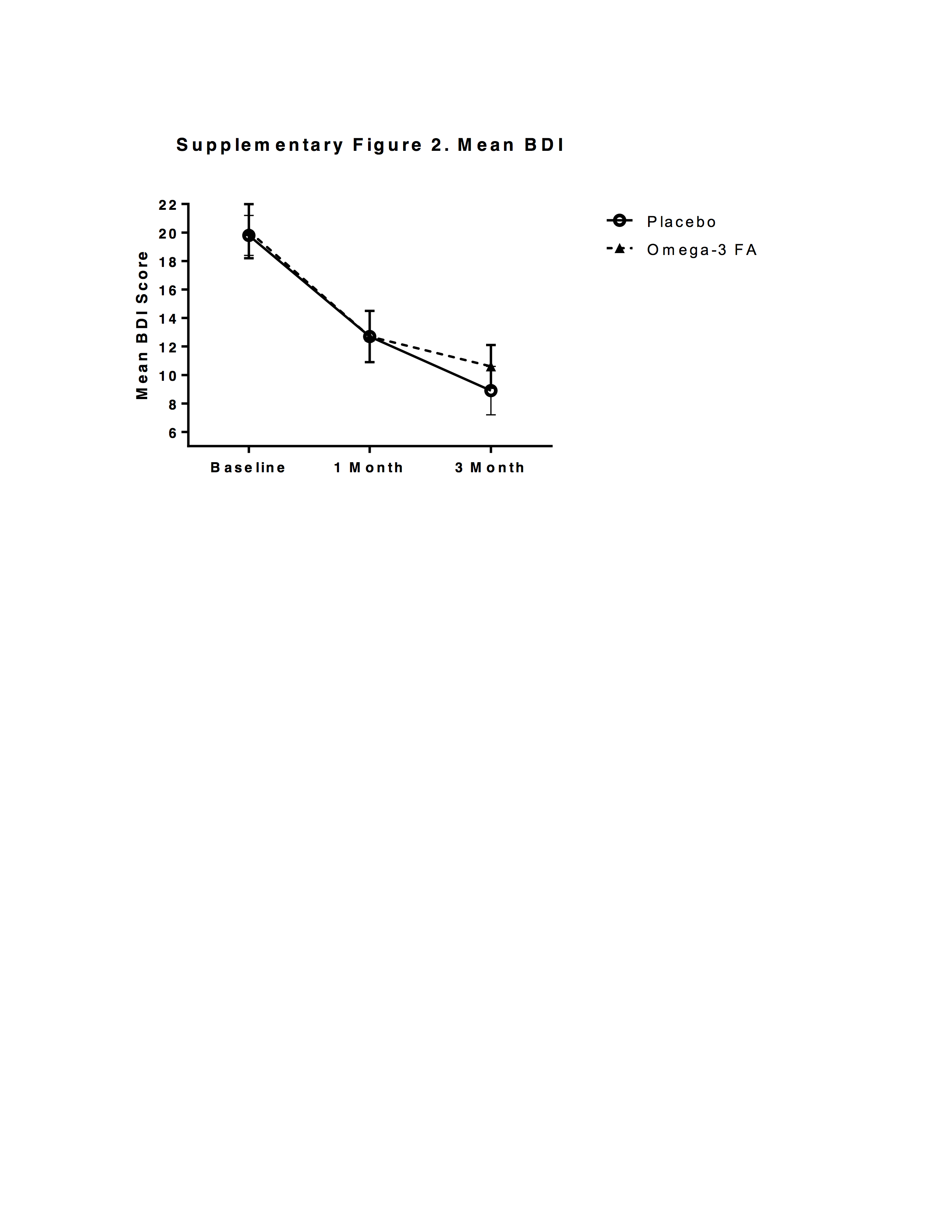

Supplement: S2 Fig — Linear mixed effects model adjusted for age and MS disease duration, error bars indicate standard error of the mean. No difference between placebo and omega-3 FA was found over 3 months (p = 0.27). (TIFF) [file pone.0147195.s004.tiff]
