# Supplementary material for: Omega-3 Fatty Acids for Depression in Multiple Sclerosis: A Randomized Pilot Study
Source: PLoS One. 2016 Jan 22;11(1):e0147195. doi: 10.1371/journal.pone.0147195 (PMC4723316; doi:10.1371/journal.pone.0147195)
Supplement: S1 Protocol — (DOC) [file pone.0147195.s005.doc]

IRB #8295, EIRB #179

Revised: 6/20/05

## Oregon Health & Science University

**RESEARCH PROTOCOL**

Protocol Title: Fish oil as an adjunct therapy for depression in multiple sclerosis

**Investigators:**

|  | **Name** | **Degrees** | **Department & Mailcode** | **Phone** | **E-Mail** |
| --- | --- | --- | --- | --- | --- |
| **Principal Investigator:** | Lynne Shinto | ND | L226 neurology | 4-5035 | shintol@ohsu.edu |
| **Co-Investigators:** | Dennis Bourdette | MD | L226 | 4-7321 | bourdett@ohsu.edu |
| **Co-Investigators:** | Gail Marracci | PhD | VA, RND65 | 095-56590 | marracci@ohsu.edu |
| **Co-Investigators:** | Peter Hauser | MD | VA, P3MHADM | 095-56490 | Peter.Hauser2@med.va.gov |
| **Co-Investigators:** | Michele Mass | MD | UHS42, neuro. | 4-5759 | massm@ohsu.edu |
| **Co-Investigators:** | Cynthia Morris | PhD, MPH | BICC, Med. Informatics clinical epi | 4-3262 | morrisc@ohsu.edu |
| **Co-Investigators:** | Rachel Frank | BA | UHS42 neuro. | 4-7963 | frankra@ohsu.edu |
| **Co-Investigators:** | Vijayshree Yadav | MD | L226 neurology | 4-5759 | yadavv@ohsu.edu |
| **Co-Investigators:** | Micha Rahder | BA | VA, RND65 | 095-56590 | [rahderm@ohsu.edu](mailto:rahderm@ohsu.edu) |
| **Co-Investigators:** | Katie Stem | BA | VA, RND65 | 095-56590 | stemk@ohsu.edu |
| **Co-Investigators:** | Debbie Griffith | RN | UHS42, neuro. | 4-7651 | griffide@ohsu.edu |
| **Co-Investigators:** | Sara Baldauf-Wagner | BA | L226 neurology | 4-3549 | baldaufw@ohsu.edu |
| **Co-Investigators:** | Ashlee Whitehead | MA | VA**,** P3MHDC | 095-57429 | Ashlee.Whitehead@med.va.gov |
| **Co-Investigator MD of Record*:**  **(if PI, so indicate)** | Dennis Bourdette | M.D. | L226 neurology | 4-7321 | bourdett@ohsu.edu |
| **Contact Person:** | Rachel Frank | BA | UHS42 neuro. | 4-7963 | frankra@ohsu.edu |

*Required by NIH if the PI is not a licensed MD with clinical privileges. Will be considered a co-investigator for GCRC reporting purposes.

**RCR Training**: Have all study personnel completed RCR training? [X] Yes [ ] Pending

(Must be complete before GCRC approval will be given.)

**Sponsor/Funding Source**: National Center for Complementary and Alternative Medicine (NCCAM)/National Institutes of Health (NIH)

# Structured Scientific Abstract

Depression is more common in MS than in many other chronic illnesses with 50-60% of MS patients affected by depression. Supplementation with omega-3 fatty acids has been reported to significantly reduced depression and to have low side effects in subjects with unipolar depression that were on stable doses of anti-depressant medication. Omega-3 fatty acid supplementation, in the form of fish oil, may offer a safe adjunctive therapy to improve the therapeutic benefits of antidepressants in MS. The primary aim of this pilot study is to determine if fish oil supplementation is an effective and safe adjunctive treatment for mild to moderate depression in people with MS. This will be a 3-month randomized, double blind, placebo controlled pilot trial of fish oil supplementation for the treatment of mild-moderately depression people with MS. Subjects in both the placebo and fish oil group that have responded to treatment (> 50% improvement on either the Montgomery-Asberg Depression Rating Scale (MADRS) or Beck Depression Inventory (BDI)) will have the option to continue on treatment for an additional 3 months to evaluate longer-term effects. Treatment during this continuation phase will remain blinded. Sixty subjects age 18-65 years old with a definite diagnosis of MS that have been diagnosed with major depressive disorder (MDD), based on the Structured Clinical Interview for DSM-IV (SCID), will be randomized to one of two groups: placebo or 6.0 g/day fish oil supplementation (2.1 g/day EPA, 1.5 g/day DHA). All subjects will maintain stable doses of their pre-study antidepressant and MS disease modifying medications while participating in the study.

Our primary objective, Aim 1, is to determine the treatment effect on depression by using the MADRS and BDI. We will also monitor adverse events by subjects self-report of general health and laboratory tests (metabolic panel, including liver function tests and prothrombin time). We will monitor worsening of depression and suicidal ideation using both depression inventories, BDI and MADRS. Our secondary objective, Aim 2, is to determine the treatment effect on inflammatory cytokine levels, as fish oils may exert its clinical effect by decreasing peripheral levels of proinflammatory cytokines. We will measure cytokines released from mitogen stimulated peripheral blood mononuclear cells (PBMCs) by immuno-assay. Compliance will be monitored by pill count and red blood cell fatty acid analysis. Data collected from this pilot study will be used to aid us in the design of a larger, multi-center clinical trial that could more fully evaluate the effectiveness of this therapy.

**A. SPECIFIC AIMS**

Aim 1) To determine if fish oil supplementation is an effective and safe adjunctive treatment for mild to moderate depression in people with MS

A. We hypothesize that 3 months of fish oil supplementation will improve depression scores on the Montgomery-Asberg depression rating scale (MADRS) or Beck Depression Inventory (BDI) more than the placebo treated group. Subjects in both the placebo and fish oil group that have responded to treatment (> 50% improvement on MADRS or BDI) will have the option to continue on treatment for an additional 3 months to evaluate longer-term effects.

B. We hypothesize that fish oil at the doses given is safe in MS subjects with mild to moderate depression taking MS disease-modifying drugs and anti-depressants. We will monitor the following: effects on blood clotting by prothrombin time (PT/INR); metabolic function including liver function by comprehensive metabolic panel; adverse events reporting (includes subject’s report of general health and screening for worsening of depression by BDI and MADRS).

Aim 2) To determine the effects of fish oil supplementation on pro-inflammatory agents related to depression and MS.

We hypothesize that one mechanism by which fish oil may exert its clinical effect is by decreasing peripheral levels of proinflammatory agents (cytokines). We expect to see a decrease in peripheral levels of inflammatory cytokines in subjects treated with fish oil.

We will measure the following cytokines release from mitogen stimulated peripheral blood mononuclear cells (PBMC) at 2 baseline time points and then at 1, 3, & 6 months of supplementation: tumor necrosis factor alpha (TNF-α), interferon gamma (IFN-γ), interleukin 2 (IL-2), interleukin-6 (IL-6), interleukin-4 (IL-4), and interleukin-10 (IL-10).

**B. BACKGROUND and SIGNIFICANCE**

It is estimated that 50-60% of patients with MS are affected with depression [1]. Although antidepressants show great benefit in alleviating depression, 29%-46% of depressed patients show a partial to no response to antidepressant therapy [2]. The treatment strategy for these patients includes increasing medication dose, switching to a different type of antidepressant medication, or augmenting current medication with an additional antidepressant [3]. All of these strategies pose safety concerns which include: lack of safety evidence for these strategies, severe side effects while switching therapies, potential for “serotonin syndrome” when combining two antidepressant agents [3]. Identifying safe and effective adjunctive therapies for depression in MS is warranted given the high prevalence of depression and the high use of anti-depressant medication in this population. The omega-3 fatty acids have been reported to improve depression when used as an adjunctive therapy in unipolar depression [4-6]. Because an increase in inflammatory mediators have now been associated with depression in MS [7, 8], omega-3 fatty acid supplementation may offer clinical benefit by decreasing peripheral levels of pro-inflammatory mediators. As fish oils possess strong anti-inflammatory properties, its adjunctive value for depression may be of special importance in inflammatory diseases in general.

#### Inflammatory cytokines may contribute to depression in MS

Major depression is associated with activation of the inflammatory response system (IRS) that includes increased numbers of leukocytes, activated T-cells and neurophils resulting in an increase in the production PGE2, TNF-α, IFN-γ, interleukin-1 beta (IL-1β), interleukin-6 (IL-6), and cytokine receptors for IL-1 (IL-1R) and interleukin-2 (IL-2) [9-18]. Although the mechanisms by which inflammatory mediators induce depression have not been elucidated, it is suggested that they may act by disrupting the hypothalamo-pituitary-adrenocortical (HPA) axis [19] or by indirectly decreasing CNS serotonin levels by catabolizing tryptophan in the periphery [20, 21]. This body of evidence linking major depression to activation of inflammatory response is of particular interest in MS as some of the same pro-inflammatory mediators implicated in major depression are implicated in MS immunopathogenesis.

There are two reports examining the association between depression, inflammatory cytokines, and MS [7, 8]. The first reported a significant positive correlation between TNF-α (r =.55) and IFN-γ (r =.54) mRNA expression levels and the BDI sum scores in MS patients. The second reported a significant positive correlation between baseline BDI score and IFN-γ production (r =.64) in 14 subjects with relapsing-remitting MS diagnosed with MDD [8].

Conventional treatments for depression in MS

Conventional treatment for depression in MS includes antidepressant medication, individual psychotherapy, and group psychotherapy. Antidepressant medication is probably the most widely used therapy for depression in MS. Very few studies evaluating the efficacy of antidepressants in MS have screened patients for major depressive disorder using DSM-IV criteria for depression [22]. One comparative study evaluating the effect of antidepressant therapy for MDD in MS reports that treatment significantly reduced depression as measured by the BDI. The study randomized 63 subjects that met criteria for both MS and major depressive disorder to one of three treatment groups: individual cognitive-behavioral therapy, supportive-expressive group therapy, or treatment with sertraline. Clinical outcomes using a 50% reduction from the baseline score of the BDI as a response criterion found that only 24% (n=5) of the sertraline group achieved significant improvements. The authors suggest that although depression in MS is responsive to treatment, it is more difficult to treat than suggested in previous studies (76% of the sertraline group did not achieve significant improvements in depression scores).

#### Omega-3 fatty acids a safe adjunctive treatment for depression

- There are three reported double-blind placebo controlled pilot studies evaluating the effects of omega-3 fatty acids in depression. All three studies report a significant improvement depression in subjects treated with omega-3 fatty acids. The first was a study of 20 patients diagnosed with MDD using DSM-IV criteria. Subjects were enrolled if they met criteria for MDD, had a baseline score on the HDRS (17 item) of > 18, were 18-75 years old, had no alcohol and drug abuse, no psychotic features, and had no comorbid psychiatric diagnosis other than obsessive-compulsive disorder, panic disorder, or dysthymic disorder [5]. Subjects maintained their pre-study doses of antidepressant medication and were randomized to one of two groups: placebo or 2g/day ethyl-eicosapentaenoic acid (E-EPA), a type of omega-3 fatty acid. After 4 weeks there was a significant improvement in mean HDRS scores for the fish oil group compared to the placebo group, E-EPA 11.6 + 6.2, placebo 20.0 + 8.8; (p<0.001). The second study was a dose-ranging pilot study on the effects of E-EPA for depression [4]. In this study 70 patients were randomized to 1 of 4 treatment groups: placebo, 1g/day E-EPA, 2 g/day E-EPA, 4 g/day E-EPA. Treatment efficacy was assessed by the HDRS (17 item), the Montgomery-Asberg Depression Rating Scale (MADRS), and the BDI at 4, 8, and 12 weeks. All subjects maintained their pre-trial doses of antidepressant medication throughout the trial. By week 12, a significant improvement in depression by all three measures was achieved in the 1g/day group compared to controls and the other two doses. The authors noted that all doses of E-EPA were well tolerated, there were no dropouts in this study, and the most common side effect in the treatment group was mild and transient gastrointestinal upset. The authors conclude that E-EPA at 1 g/day significantly improves depression in people that are currently taking antidepressants. The investigators note that although their study found efficacy at the 1-g/day dose, other patients may require higher doses of E-EPA.

The third study used methodology similar to the first study except that subject’s received either placebo oil or fish oil (five 1-gram capsules per day containing 2.20 g EPA and 1.10 g docosahexanoic acid (DHA)). The HDRS was used to assess depression. After 8 weeks of treatment there was a significant difference in mean HDRS, placebo 15.7 + 3.7 and fish oil 8.9 + 3.2 (p<0.001) [6].

#### Omega-3 fatty acids possess anti-inflammatory actions

The immunomodulatory effects of omega-3 fatty acids have been well documented in scientific studies*. In vitro*, animal, and, *ex vivo* human studies have reported a decrease in mRNA and protein levels of a number of cytokines including TNF-α, IFN-γ, IL-1, IL-2, VCAM-1 [23]. A single published study reported a significant decrease, from baseline, in the levels of IL-1β (p<0.03), TNF-α (p<0.02), IL-2 (p<0.002), and IFN-γ (p<0.01) produced from unstimulated and stimulated PBMC of twenty MS subjects and fifteen healthy controls after three months supplementation with fish oil [24].

Conclusions

Antidepressant medication is a widely used and effective treatment for depression in MS; however, even with treatment many MS patients still experience mild to moderate depression. The same pro-inflammatory cytokines involved in MS disease pathogenesis are now implicated in the pathogenesis of major depressive disorder. Fish oil supplementation is reported in a number of studies, including one MS study, to decrease levels of peripheral pro-inflammatory cytokines and may exert its clinical effect by this mechanism. Fish oil may offer a safe and effective adjunctive therapy for depression in MS patients.

# C. PRELIMINARY STUDIES/PROGRESS REPORT

We have baseline data on depression scores from the BDI from 37 subjects that had participated in a naturopathic treatment pilot study in MS. Of the 37 subjects, 44% (16/37) were on antidepressants and 84% (29/37) were on one of the MS disease modifying medications. Of the 16 subjects taking antidepressants 7 subjects scored > 17 on the BDI. We compared this group with a group of 9 subjects taking anti-depressants that scored <17 on the BDI-21, mean = 6.4, s.d. + 4.2 and found that the group mean scores were significantly different, p=0.002. These results indicate that 44% (7/16) of study subjects on antidepressant therapy are still scoring significantly high on the BDI.

# D. RESEARCH DESIGN and METHODS

#### *Overall study design*

Phase I of this study will be a 3-month randomized, double blind, placebo controlled pilot trial. There will be 7 visits over the course of 3-months. Sixty subjects age 18-65 years old with a definite diagnosis of MS [25] that have been diagnosed with MDD, based on the Structured Clinical Interview for DSM-IV (SCID), will be randomized to one of two groups: placebo or 6.0 g/day fish oil supplementation (2.1 g/day EPA, 1.5 g/day DHA). All subjects will maintain stable doses of their pre-study antidepressant and MS disease modifying medications while participating in the study. Subjects in both the placebo and fish oil group that have responded to treatment (> 50% improvement on MADRS or BDI) will have the option to continue on treatment for an additional 3 months to evaluate longer-term effects, Phase II of this study. There will be 3 visits during Phase II and treatment during this phase will remain blinded. Clinical and biological measures will occur at baseline and 1, 3 & 6 months of supplementation.

#### **Study visits**

#### Prescreening (phone contact)

All potential subjects will be prescreened by a telephone interview. The potential subject will be asked about whether or not they meet the study inclusion and exclusion criteria. Those who meet preliminary inclusion by the phone screen will be scheduled for an in-clinic screening visit at OHSU.

#### Visit 1 (Screen)

The screening visit is to establish eligibility for study participation. This visit and all subsequent visits will take place at the MS Center of Oregon at OHSU or at the General Clinical Research Center (GCRC) at OHSU. Each potential study subject will be consented at this visit after which a family and medical history will be taken. The subject will be screened for major depressive disorder using the SCID, dementia by the MMSE, and for mild to moderate depression using the MADRS. They will then be asked to fill out the BDI. Concomitant medications will also be recorded at the screening visit (*See Table 1: Study Schedule: Phase I*).

Visit 2 (Baseline)

Subjects that meet study inclusion after the screening visit will be scheduled for follow up within 2 weeks. At this visit subjects will receive a brief physical exam and a neurological exam (EDSS) from Dr. Shinto. The subject will be given a MADRS score. A research assistant will administer the MS Functional Composite (MSFC) and record concomitant medications. The subject will fill out study questionnaires which include: BDI, Multiple Sclerosis Quality of Life Inventory (MSQLI), and Food Frequency. A research assistant will draw blood for cytokine levels, prothrombin time (PT/INR), comprehensive metabolic panel, and RBC fatty acid analysis.

#### Visit 3 (day 0)

#### This visit will occur one week after baseline. At this visit, subjects will receive study medication for 1 month and be instructed on the dose.

#### Visit 4 (week 2)

At this visit subjects will be screened for worsening of depression (>30 on the BDI) and suicidal ideation using question #9 from the BDI. If either of these occurs the subject will be immediately referred for standard care and will be exited from the study. General health adverse events will also be recorded at this visit. Dr. Shinto will be available by pager 24 hours a day to address patient safety issues. Dr. Hauser will be available to discuss these cases with Dr. Shinto. The subject will be instructed to bring their medication bottle to their next scheduled visit.

Visit 5 (week 4)

All measures except PE/neurological exam/EDSS, the Food Frequency questionnaire, and the MSFC will be assessed at this visit. A research assistant will draw blood for prothrombin time (PT/INR), comprehensive metabolic panel, cytokine levels, and RBC fatty acid analysis. A research assistant will assess the subject for adverse events and perform a pill count. The subject will also be asked about any changes in medication or doses of medications (prescribed and over the counter). The subject will be instructed to bring their medication bottle to their next scheduled visit.

#### Visit 6 (week 8)

MADRS and BDI will be administered. A research assistant will assess the subject for adverse events and perform a pill count. The subject will also be asked about any changes in medication

or doses of medications (prescribed and over the counter). The subject will be instructed to bring their medication bottle to their next scheduled visit.

Visit 7 (week 12)

This visit will assess all of the same measures as Visit 2. In addition, the subject will return all remaining study drug and a pill count will be performed. If the subject has achieved a 50% improvement on their BDI or MADRS score they may choose to continue on in the study for another 3 months or to exit the study. If the subject chooses to remain in the study, we will review the consent form for Phase II of the study and attain consent before proceeding with Phase II. After being consented the subject for the subject will receive another month’s supply of study medication and will be instructed to bring this bottle to their next scheduled visit.

Table 1. Study Schedule: Phase I

| **Assessments** | **Visit 1**  Screen | **Visit 2**  Baseline | **Visit 3**  Wk 0 | Phone  Wk 1 | **Visit 4**  Wk 2 | Phone  Wk 3 | **Visit 5**  Wk 4 | **Visit 6**  Wk 8 | Phone  Wk 10 | **Visit 7**  Wk 12 |
| --- | --- | --- | --- | --- | --- | --- | --- | --- | --- | --- |
| Family and Medical Hx | X |  |  |  |  |  |  |  |  |  |
| PE/Neurological exam/EDSS |  | X |  |  |  |  |  |  |  | X |
| SCID | X |  |  |  |  |  |  |  |  |  |
| BDI | X | X |  | X | X | X | X | X | X | X |
| MADRS | X | X |  |  |  |  | X | X |  | X |
| MMSE | X |  |  |  |  |  |  |  |  |  |
| MSQLI |  | X |  |  |  |  | X |  |  | X |
| MSFC |  | X |  |  |  |  |  |  |  | X |
| Cytokine measurement |  | X |  |  |  |  | X |  |  | X |
| Metabolic panel, PT time |  | X |  |  |  |  | X |  |  | X |
| RBC fatty acid |  | X |  |  |  |  | X |  |  | X |
| Food freq. survey |  | X |  |  |  |  |  |  |  | X |
| Concomitant medications | X | X |  |  |  |  | X | X |  | X |
| Adverse Events |  |  | X | X | X | X | X | X | X | X |
| Pill Count |  |  |  |  |  |  | X | X |  | X |
| Blinding evaluation |  |  |  |  |  |  |  |  |  | X |

Table 2. Study Schedule: Phase II

| **Assessments** | Month 4 | | Month 5 | | Month 6 | |
| --- | --- | --- | --- | --- | --- | --- |
|  | Phone  wk  14 | Visit 8 wk  16 | phone  wk  18 | Visit 9 wk  20 | phone  wk  22 | Visit 10 wk  24 |
| Family and Medical History |  |  |  |  |  |  |
| PE/Neurological exam/EDSS |  |  |  |  |  | X |
| SCID |  |  |  |  |  | X |
| MADRS |  | X |  | X |  | X |
| BDI | X | X | X | X | X | X |
| MSQLI |  |  |  |  |  | X |
| MSFC |  |  |  |  |  | X |
| Cytokine measurement |  |  |  |  |  | X |
| Metabolic panel, PT |  |  |  |  |  | X |
| RBC fatty acid |  |  |  |  |  | X |
| Food freq. survey |  |  |  |  |  | X |
| Concomitant medications |  | X |  | X |  | X |
| Adverse Events | X | X | X | X | X | X |
| Pill Count |  | X |  | X |  | X |
| Blinding evaluation |  |  |  |  |  | X |

#### Phase II: Visits 8(week 16) and Visit 9 (week 20)

Subjects will be come into the clinic to be assessed for MADRS and BDI. A research assistant will assess the subject for adverse events and perform a pill count. The subject will also be asked about any changes in medication or doses of medications (prescribed and over the counter). (**See Table 2: Study Schedule: Phase II**)

Phase II: Visit 10 (week 24)

The subject will be re-screened for MDD using the SCID and attain a MADRS score. Dr. Shinto will perform a brief PE and a neurological exam (EDSS). A research assistant will administer the MSFC and record concomitant medications. The subject will fill out study questionnaires, which include: BDI, MSQLI, Food Frequency. A research assistant will draw blood for comprehensive metabolic function, PT/INR, cytokine levels, and RBC fatty acid analysis. Adverse events, pill count, and concomitant medications will be recorded at this visit. A short questionnaire that assesses subject blinding will be administered at this visit.

#### Study dose

We are using doses and ratios of EPA and DHA similar to those reported to improve depression in people on stable doses of antidepressant medication [6]. The subjects randomized to the fish oil group will receive 6.0 grams of fish oil per day providing 2.1 g/day of EPA and 1.5g/day of DHA. Subjects will take 3 capsules twice a day with meals. The placebo capsules will contain soybean oil with 1% fish oil so that it will be flavored to taste and smell similar to the fish oil capsules. The placebo group will receive 6 placebo capsules per day and will take 3 capsules twice a day with meals.

# Product quality control

Nordic Naturals will supply the fish oil supplement. This company follows the FDA guidelines for Good Manufacturing Procedures and verifies product quality and label claim by in-house and outside lab analysis. The fish oil capsules are tested for contaminants (e.g. heavy metals), lipid oxidation, and for omega-3 fatty acid content.

Analysis of outcome variables

The major outcome measures in this study are the 3-month change in MADRS and IFN-γ. Initially we will compare the difference in MADRS scores, BDI scores, and IFN-γ levels between the fish oil and placebo groups using a two-sample t-test. In a more exploratory analysis, we will then assess whether the change in IFN-γ levels is correlated with the change in MADRS. To do this we will build a multiple regression model, where the predictors are the baseline MADRS or BDI and IFN-γ levels and the change in IFN-γ levels. We will additionally assess whether there is an interaction between baseline IFN-γ and fish oil supplementation by extending the above regression to include the interactions terms with fish oil. In order to control for potential confounders (specifically age, gender, education level, disability, and fatigue), variable selection schemes (all possible regressions using maximum R^2^ and backwards elimination of variable) will be used to determine if any of these potential confounders predict the dependent variable. If so, these potential confounders will be included in the regression models as covariates.

#### Statistical Analysis Statistical power and sample size

The study that used 2g/day of E-EPA reported a 9.3-unit difference between baseline and 4-week differences in placebo and active groups for the HDRS, this difference was statistically significant (p < 0.001) [5]. The standard deviation for the groups was 7.4 (the larger of the standard deviations). A sample size of 11 subjects per group provides 80% power with an alpha= 0.05, to detect the above difference in HDRS between the treatment and placebo group. A sample size of 18 subjects per group will provide power greater than 95% with an alpha= 0.05, to detect a difference of 500 units for IFN-γ levels between baseline and post-treatment levels. This assumes a standard deviation of 400 units and is based on the results of a study that reported a decrease in IFN-γ levels in MS patients with MDD from baseline values after 16-wks of either therapy based or medication based treatments for depression [8]. Assuming a dropout rate of 20% the sample size of 60 subjects, or 30 per group is also more than adequate to detect a treatment difference in IFN-γ levels produced from PBMC. The additional sample will provide additional power for the exploratory analyses using the regression analysis. We are using the MADRS as the primary outcome measure as it is highly correlated with the HDRS [26-28] and will be more sensitive to treatment change in patients with MS than the HDRS. As we expect MADRS scores to have a greater change with treatment than the HDRS, we are well powered with 60 subjects.

#### Randomization

After study eligibility is established subjects will be randomized to placebo or fish oil supplementation by using a computer-generated scheme. Because of sample size limitations we will only stratify on one parameter prior to randomization, which will be the BDI score at screening. Subjects will be stratified into two categories “mildly depressed” with BDI scores 10-17 and “moderately depressed” with BDI scores 18-30. This will insure that each group will contain equal numbers of mild and moderately depressed subjects. The data management coordinator that is in charge of labeling medication will maintain the randomization list.

#### Compliance and Adverse Events

Compliance will be measured by pill count and RBC fatty acid analysis. Refer to section E. Human Subjects, “protection against risks”, p. 14 for monitoring of adverse events.

Blinding, Data Collection and Management

Refer to section E. Human Subjects, “sources of material”, p. 13.

**Assessments**

Beck Depression Inventory (BDI)

The BDI is a 21-item self-report measure of depression [30] and is commonly used in MS studies [7, 8, 22, 31, 32]. It is an easily administered measure for depression that is not clinician-dependent. We will examine how well the BDI scores correlate with clinician rated scores of depression ( MADRS).

Structured Clinical Interview for DSM-IV (SCID)

The SCID is a semi-structured interview that can be administered by a well-trained clinician or research associate. It is designed to assess major Axis I disorders [31] and will be used to assess MDD at screening and after 3 months and 6 months on supplementation.

The Montgomery-Asberg Depression Rating Scale (MADRS)

The MADRS is a structured interview assessment of depression, designed to be especially sensitive to changes in patients’ depression symptoms after antidepressant therapy [48]. The MADRS scale is more oriented towards psychic rather than somatic symptoms of depression [47].

Expanded Disability Status Scale (EDSS)

The EDSS is an ordinal scale giving a measure of neurological impairment on a scale between 0 (normal) to 10.0 (dead). Patients with scores in the .5-4.0 range have mild disability and are able to walk at least 500 meters without aid or rest. Patients with scores of > 7.5 are cannot walk and have upper extremity dysfunction [32].

Multiple Sclerosis Functional Composite (MSFC)

The MSFC includes measurements of ambulation (timed 25 foot walk), upper extremity function (nine hole peg test) and cognitive performance (PASAT III) [33]. It is an objective measure of disability and is often used in MS studies along with the EDSS to evaluate treatment effect on disability.

Mini-Mental State Exam (MMSE)

The MMSE is among the most widely used screening instruments for cognitive impairment indicative of dementia[34]. This 30-point scale is helpful for comparing across diverse studies as it has become a common way of assessing severity of dementia among clinicians. Any subject that scores 24 or less on the MMSE indicates a mild to severe forms of dementia. As dementia can significantly affect depression scores we will screen for and exclude subjects with dementia.

Multiple Sclerosis Quality of Life Inventory (MSQLI)

The MSQLI is a modular MS-specific HRQL instrument consisting of the SF-36 and supplemented by nine symptom specific measures (fatigue, pain, bladder function, bowel function, emotional status, perceived cognitive function, visual function, sexual satisfaction, and social relationships). The reliability and construct validity of this measure has been evaluated by a field test of MS patients with a broad range of disability (EDSS 0-8.5), and its content has been validated by MS specialists (neurologists and allied health professionals), HRQL experts, patients and caregivers [35]. A modified version of the FIS is incorporated into the MSQLI. The FIS is a well-validated measure of fatigue[36, 37].

#### Block Food Frequency Questionnaire

The Block food frequency questionnaire [38]. This 147-item instrument is based on extensive population surveys validated by food records.

#### RBC fatty acid

RBC analysis is a quantitative measure of the incorporation of fatty acids into cell membranes. This assay will be performed in the Lipid laboratory at OHSU. These methods have been validated and previously published [39, 40].

#### Peripheral Blood Mononuclear Cell (PBMC) Separation

PBMC will be isolated from heparinized blood within 4 hours of blood draw (Ficoll Hypaque; Pharmacia Biotech, Uppsala, Sweden). This method of separation has been described in previously published reports [41, 42]. Separated PBMCs will be stored at –80°C for cytokine measurement. PMBCs will also be frozen and stored for future testing of gene expression by microarray assay.

#### Stimulation of PBMC

PBMC will be stimulated with a mitogen (Concanavalin A). One ml of freshly isolated PBMC suspension will be added to each well. Cells will be cultured for 48 hours at 37°C and 7% CO_2_, after which time cultures will be clarified by centrifugation and supernatants were aliquoted and frozen at –80°C until used for cytometric bead array analyses.

#### Cytokine measurement

Cytokine levels from unstimulated and stimulated PBMC will be measured using the Cytometric Bead Assay (CBA, BD Biosciences, Pharmigen). The company reports good intra- and inter- assay reproducibility for all cytokines measured: Intra-assay %CV < 5.0; Inter-assay % CV (< 80 pg/ml) < 11.0; Inter-assay %CV (625-2500 pg/ml) < 6%. The CBA kit was chosen to measure cytokines because it a reliable, precise, and economical method for measuring an array of human cytokines in a single sample.

#### Other laboratory tests

Comprehensive metabolic panel that includes liver function tests and prothrombin time will be determined in the laboratory at OHSU. We will use these measures along with reports of adverse events to monitor treatment safety. Matrixmetalloproteinase-9 levels from stimulated PBMC will be measured by ELISA, as they mediators of the inflammatory process by allowing inflammatory cells into cross the blood brain barrier.

# E. HUMAN SUBJECTS

1. ***Human Subjects Involvement and Characteristics***

A total of 60 subjects will be enrolled in this trial. We will select subjects with relapsing remitting MS that have mild to moderate depression. Women and members of minority groups will be included.

#### **Inclusion Criteria**

-Definite MS by McDonald criteria [25]

-Relapsing remitting course of MS

-Meets DSM-IV criteria based on SCID for Axis I disorders [31]

-Age 18 or older

- Scores between 11-30 on the MADRS (indicating mild to moderate depression) [26]

-Scores 25 or greater on the MMSE

-no essential fatty acid supplementation and < 6 oz per week of fish in diet at least 1 month prior to enrollment

-on stable dose of anti-depressant medication (no change in medication or dose at least 3 months prior to enrollment)

-on stable dose of interferon beta or glatiramer acetate (no change in MS disease modifying medication at least 6 months prior to enrollment)

-Able to understand and sign consent

#### **Exclusion Criteria**

-Severe depression with suicidal ideation and plan as determined by SCID >20 and MADRS > 30

-Serious psychological disorders (e.g. psychotic disorders, bipolar disorder)

-Taking 2 or more antidepressants

-Plans to change antidepressant therapy within 3 months of enrollment

-Serious medical conditions such as congestive heart failure, cancer, liver disease, kidney failure.

-Current or past history of clinically significant ventricular arrythmias: includes patients that are receiving implantable cardioverter defibrillator (ICD) for sustained ventricular tachycardia (VT) or ventricular fibrillation (VF), or patients that had received an ICD or ICD therapy for an episode of VT/VF within the past 6 months, or subjects currently taking anti-arrythmia medications for VT/VF (e.g. Quinidine, Amiodarone).

-MS exacerbation or corticosteroid treatment 1 month prior to enrollment

-Pregnancy (pregnancy might alter results on outcome measures)

-Enrolled in another study

Because of the short duration of this study, subject participation will be stopped for the following reasons: MS exacerbation, change in MS disease modifying medication, and change or increase in antidepressant medication.

***b) Rationale for inclusion of vulnerable populations:***

NA. While MS patients are considered ill they are not considered a vulnerable population.

***c) Sources of Materials:***

This is a double-blind placebo controlled study. The study investigators, research associates involved in subject assessment, and subjects will have no knowledge of study assignment. Data analysis will be performed blinded to treatment status. The General Clinical Research Center (GCRC) at OHSU houses a biostatistics resource center that will create the randomization scheme and will ensure blinding of all study medications and data records. Study medication will be handled, labeled, and distributed by pharmacists in the Investigational Drug Service who are not involved in either the outcome assessments or data analysis. We will also evaluate the effectiveness of our blinding by giving both study evaluators and subjects a short questionnaire asking about knowledge of group assignment. If a subject reports serious side effects related to the study medication and information on group assignment is believed to be necessary for the subject’s safety and well being, the blind will be broken for that subject. The randomization code will otherwise be broken only after data analysis or if there are numerous reports of serious adverse events before the end of the study. To insure confidentiality, all data sources will be coded with a unique identification number that has no personal identifying information. All study files will be stored in locked cabinets in the trial coordinator’s office. Computer data will be protected with passwords known only to the appropriate staff. All data obtained from this study will be used for research purposes only.

Data will be collected at eight points during the course of the study.

***d) Potential Risks:***

The risks to subjects participating in this study are minimal. The risks may include mild anxiety from filling out questionnaires, and mild side effects from fish oil or placebo capsules. Subjects may feel some pain when blood is drawn. There is a small chance the needle will cause bleeding, a bruise, or an infection. There have been few reports of adverse events in people taking up to 15 grams of fish oil per day [45]; we do not anticipate serious adverse events from fish oil supplementation using a dose of 6.0 grams per day. Because fish oil does have antiplatelet and antithromboxane A_2_  levels, there is a concern about increase bleeding time and interactions with blood thinning medications (e.g. warfarin). However, a study evaluating the effects of 1 year of fish oil supplementation on patients undergoing artery bypass reported no increase in bleeding during surgery in patients given fish oil plus aspirin or given fish oil plus warfarin compared to patients given those therapies alone [46]. In one double-blind, placebo-controlled trial of people with current or past history of ventricular tachycardia or ventricular fibrillation receiving implantable cardioverter defibrillator (ICD) or patients that had received an ICD or ICD therapy for an episode of VT/VF, it was reported that the group receiving fish oil at 1.8 grams/day had an increase risk of VT or VF compared to those receiving placebo [49]. Because of this potential risk, we are excluding patients with this condition and patients taking anti-arrhythmia medication from the study.

***e) Recruitment and Informed Consent:***

Recruitment will occur after the Institutional Review Board at OHSU approves the study.

Subjects with MS will be recruited from the greater Portland metropolitan area in the form of mailings to MS Center of Oregon patients, mailings to neurologists, advertisements on the MS Center of Oregon website and in the newsletter of the Oregon chapter of the National MS Society (NMSS). The Oregon Chapter of the NMSS has a statewide mailing list of over 5,000 members and has assisted the MS Center in recruitment for both CAM and conventional intervention studies. Consent will be obtained during the screening visit, which will occur at the MS Center of Oregon. Dr. Shinto or a research assistant will go over the entire consent form with the potential subject and will be available to answer any questions. Dr. Shinto or the research assistant will then ask the potential subject if they understand the purpose of the study and the risks and benefits associated with the study. The potential subject will have the opportunity to take the informed consent home to review. No testing or examinations will be performed before the consent form is understood and signed. A second informed consent will be obtained in the same fashion from subjects who qualify and are willing to participate in the continuation phase of the study.

*f) Protection against risk:*

The Data Safety and Monitoring Board of ORCCAMIND will monitor this study to ensure the safety of subjects. We will monitor adverse events by laboratory tests, which include prothrombin time (PT/INR) and comprehensive metabolic panel. For the first month of the study general health events, worsening of depression (>30 on BDI), and suicidal ideation (question # 9 on BDI) will be monitored weekly (2 x by phone and 1x in-clinic) for all subjects. If either of these occurs the subject will be immediately referred for standard care and will be exited from the study. Dr. Shinto will be available by pager 24 hours a day to address patient safety issues. Dr. Hauser will be available to discuss these cases with Dr. Shinto. After the first month, subjects will be monitored every other week for general health events, worsening of depression (>30 on BDI), and suicidal ideation (question # 9 on BDI) (1 x by phone and 1x in-clinic). The same procedures described for month-1 will apply for all subjects throughout the study period. Subjects that have an acute MS exacerbation during the study will be referred to their neurologist for care.

***g) Potential Benefits of the Proposed Research to the Subjects and Others:***

Identifying safe and effective therapies that can decrease depression for those that are not responding well to antidepressant medication is of potential benefit for people with MS. In addition, fish oils have anti-inflammatory properties that may also have potential benefits for MS, which is an inflammatory condition of the CNS. Fish oils have also been reported to decrease triglyceride levels, so this therapy may be very beneficial to subjects that are at risk for cardiovascular disease.

**h)** ***Importance of Knowledge to be Gained:***

Complementary and alternative medicine (CAM) use is high in people with MS, yet there is little scientific evidence on the safety and effectiveness of CAM in this population. This is a pilot study aimed at collecting preliminary data on the safety and effectiveness of fish oil as an adjunct therapy for depression in MS. Data collected from this study will aid in the construction of a larger double-blind placebo controlled trial designed so we can better evaluate the effects of fish oil supplementation in depression and better understand the mechanism by which fish oil may exert its clinical effects.

**G. LITERATURE CITED**

1. Sadovnick, AD, Remick, RA, Allen, J, et al. Depression and multiple sclerosis*.* Neurology. 1996; 46(3): 628-32

2. Fava, M,Davidson, KG. Definition and epidemiology of treatment-resistant depression*.* Psychiatr Clin North Am. 1996; 19(2): 179-200

3. Hirschfeld, RM, Montgomery, SA, Aguglia, E, et al. Partial response and nonresponse to antidepressant therapy: current approaches and treatment options*.* J Clin Psychiatry. 2002; 63(9): 826-37

4. Peet, M,Horrobin, DF. A dose-ranging study of the effects of ethyl-eicosapentaenoate in patients with ongoing depression despite apparently adequate treatment with standard drugs*.* Arch Gen Psychiatry. 2002; 59(10): 913-9

5. Nemets, B, Stahl, Z,Belmaker, RH. Addition of omega-3 fatty acid to maintenance medication treatment for recurrent unipolar depressive disorder*.* Am J Psychiatry. 2002; 159(3): 477-9

6. Su, KP, Huang, SY, Chiu, CC, et al. Omega-3 fatty acids in major depressive disorder. A preliminary double-blind, placebo-controlled trial*.* Eur Neuropsychopharmacol. 2003; 13(4): 267-71

7. Kahl, KG, Kruse, N, Faller, H, et al. Expression of tumor necrosis factor-alpha and interferon-gamma mRNA in blood cells correlates with depression scores during an acute attack in patients with multiple sclerosis*.* Psychoneuroendocrinology. 2002; 27(6): 671-81

8. Mohr, DC, Goodkin, DE, Islar, J, et al. Treatment of depression is associated with suppression of nonspecific and antigen-specific T(H)1 responses in multiple sclerosis*.* Arch Neurol. 2001; 58(7): 1081-6

9. Mikova, O, Yakimova, R, Bosmans, E, et al. Increased serum tumor necrosis factor alpha concentrations in major depression and multiple sclerosis*.* Eur Neuropsychopharmacol. 2001; 11(3): 203-8

10. Sluzewska, A, Rybakowski, J, Bosmans, E, et al. Indicators of immune activation in major depression*.* Psychiatry Res. 1996; 64(3): 161-7

11. Sluzewska, A, Rybakowski, JK, Sobieska, M, et al. Concentration and microheterogeneity glycophorms of alpha-1-acid glycoprotein in major depressive disorder*.* J Affect Disord. 1996; 39(2): 149-55

12. Seidel, A, Arolt, V, Hunstiger, M, et al. Cytokine production and serum proteins in depression*.* Scand J Immunol. 1995; 41(6): 534-8

13. Song, C, Dinan, T,Leonard, BE. Changes in immunoglobulin, complement and acute phase protein levels in the depressed patients and normal controls*.* J Affect Disord. 1994; 30(4): 283-8

14. McAdams, C,Leonard, BE. Neutrophil and monocyte phagocytosis in depressed patients*.* Prog Neuropsychopharmacol Biol Psychiatry. 1993; 17(6): 971-84

15. Maes, M, Christophe, A, Delanghe, J, et al. Lowered omega3 polyunsaturated fatty acids in serum phospholipids and cholesteryl esters of depressed patients*.* Psychiatry Res. 1999; 85(3): 275-91

16. Maes, M, Meltzer, HY, Bosmans, E, et al. Increased plasma concentrations of interleukin-6, soluble interleukin-6, soluble interleukin-2 and transferrin receptor in major depression*.* J Affect Disord. 1995; 34(4): 301-9

17. Maes, M, Bosmans, E, Suy, E, et al. Depression-related disturbances in mitogen-induced lymphocyte responses and interleukin-1 beta and soluble interleukin-2 receptor production*.* Acta Psychiatr Scand. 1991; 84(4): 379-86

18. Calabrese, JR, Skwerer, RG, Barna, B, et al. Depression, immunocompetence, and prostaglandins of the E series*.* Psychiatry Res. 1986; 17(1): 41-7

19. Leonard, BE. The immune system, depression and the action of antidepressants*.* Prog Neuropsychopharmacol Biol Psychiatry. 2001; 25(4): 767-80

20. Bonaccorso, S, Marino, V, Puzella, A, et al. Increased depressive ratings in patients with hepatitis C receiving interferon-alpha-based immunotherapy are related to interferon-alpha-induced changes in the serotonergic system*.* J Clin Psychopharmacol. 2002; 22(1): 86-90

21. Lestage, J, Verrier, D, Palin, K, et al. The enzyme indoleamine 2,3-dioxygenase is induced in the mouse brain in response to peripheral administration of lipopolysaccharide and superantigen*.* Brain Behav Immun. 2002; 16(5): 596-601

22. Mohr, DC, Boudewyn, AC, Goodkin, DE, et al. Comparative outcomes for individual cognitive-behavior therapy, supportive-expressive group psychotherapy, and sertraline for the treatment of depression in multiple sclerosis*.* J Consult Clin Psychol. 2001; 69(6): 942-9

23. Calder, PC. Dietary modification of inflammation with lipids*.* Proc Nutr Soc. 2002; 61(3): 345-58

24. Gallai, V, Sarchielli, P, Trequattrini, A, et al. Cytokine secretion and eicosanoid production in the peripheral blood mononuclear cells of MS patients undergoing dietary supplementation with n-3 polyunsaturated fatty acids*.* J Neuroimmunol. 1995; 56(2): 143-53

25. McDonald, W, Compston, A, Edan, G, et al. Recommended diagnostic criteria for multiple sclerosis: Guidelines from the international panel on the diagnosis of multiple sclerosis*.* Annals of Neurology. 2001; 50(1): 121-127

26. Williams, JB. A structured interview guide for the Hamilton Depression Rating Scale*.* Arch Gen Psychiatry. 1988; 45(8): 742-7

27. Barrett, JE, Williams, JW, Jr., Oxman, TE, et al. Treatment of dysthymia and minor depression in primary care: a randomized trial in patients aged 18 to 59 years*.* J Fam Pract. 2001; 50(5): 405-12

28. Beck, A, Ward, CH, Medelson, M, Mock, J. An inventory for measuring depression*.* Archives of General Psychiatry. 1961(4): 561-571

29. Aikens, JE, Reinecke, MA, Pliskin, NH, et al. Assessing depressive symptoms in multiple sclerosis: is it necessary to omit items from the original Beck Depression Inventory? J Behav Med. 1999; 22(2): 127-42

30. Pujol, J, Bello, J, Deus, J, et al. Lesions in the left arcuate fasciculus region and depressive symptoms in multiple sclerosis*.* Neurology. 1997; 49(4): 1105-10

31. First, M, Spitzer, RL. Structured clinical interview for DSM-IV Axis I Disorders: clinician version (SCID-CV). 1997, Washington DC: American Psychiatric Press.

32. Kurtzke, J. Rating neurological impairment in multiple sclerosis: an expanded disability status scale (EDSS). Neurology. 1983; 33: 1444-1452

33. Fischer, JS, Rudick, RA, Cutter, GR, et al. The Multiple Sclerosis Functional Composite Measure (MSFC): an integrated approach to MS clinical outcome assessment. National MS Society Clinical Outcomes Assessment Task Force*.* Mult Scler. 1999; 5(4): 244-50

34. Folstein, MF, Folstein, SE,McHugh, PR. "Mini-mental state". A practical method for grading the cognitive state of patients for the clinician*.* J Psychiatr Res. 1975; 12(3): 189-98

35. Fischer, JS, LaRocca, NG, Miller, DM, et al. Recent developments in the assessment of quality of life in multiple sclerosis (MS)*.* Mult Scler. 1999; 5(4): 251-9

36. Fisk, JD, Ritvo, PG, Ross, L, et al. Measuring the functional impact of fatigue: initial validation of the fatigue impact scale*.* Clin Infect Dis. 1994; 18 Suppl 1: S79-83

37. Fisk, JD, Pontefract, A, Ritvo, PG, et al. The impact of fatigue on patients with multiple sclerosis*.* Can J Neurol Sci. 1994; 21(1): 9-14

38. Block, G, Coyle, LM, Hartman, AM, et al. Revision of dietary analysis software for the Health Habits and History Questionnaire*.* Am J Epidemiol. 1994; 139(12): 1190-6

39. Ruyle, M, Connor, WE, Anderson, GJ, et al. Placental transfer of essential fatty acids in humans: venous-arterial difference for docosahexaenoic acid in fetal umbilical erythrocytes*.* Proc Natl Acad Sci U S A. 1990; 87(20): 7902-6

40. Anderson, GJ, Connor, WE, Corliss, JD, et al. Rapid modulation of the n-3 docosahexaenoic acid levels in the brain and retina of the newly hatched chick*.* J Lipid Res. 1989; 30(3): 433-41

41. Vandenbark, AA, Finn, T, Barnes, D, et al. Diminished frequency of interleukin-10-secreting, T-cell receptor peptide-reactive T cells in multiple sclerosis patients might allow expansion of activated memory T cells bearing the cognate BV gene*.* J Neurosci Res. 2001; 66(2): 171-6.

42. Burrows, GG, Chou, YK, Wang, C, et al. Rudimentary TCR signaling triggers default IL-10 secretion by human Th1 cells*.* J Immunol. 2001; 167(8): 4386-95.

43. Hamilton, M. A rating scale for depression*.* J Neurol Neurosurg Psychiatry. 1960; 23: 56-62

44. Beck, A, Steer, RA, Brown, GK, *BDI-II Manual*. 1996, Psychological Corporation: New York.

45. Payne, A. Nutrition and diet in the clinical management of multiple sclerosis*.* J Hum Nutr Diet. 2001; 14(5): 349-57

46. Eritsland, J, Arnesen, H, Gronseth, K, et al. Effect of dietary supplementation with n-3 fatty acids on coronary artery bypass graft patency*.* Am J Cardiol. 1996; 77(1): 31-6

47. Galinowski, A, Lehert, P. Structural validity of MADRS during antidepressant treatment. Int Clin Psychopharmacol. 1995 Sep; 10(3): 157-61.

48. Zimmerman, M, Posternak, MA, Chelminski, I. Derivation of a definition of remission on the Montgomery-Asberg depression rating scale corresponding to the definition of remission on the Hamilton rating scale for depression. J Psychiatr Res. 2004 Nov-Dec; 38(6):577-82.

49. Raitt, MH, Connor, WE, Morris, C, et al. Fish Oil Supplementation and Risk of Ventricular Tachycardia and Ventricular Fibrillation in Patients With Implantable Defibrillators: A Randomized Controlled Trial. JAMA 2005;293(23): 288491.
