# Supplementary material for: Omega-3 Fatty Acids for Depression in Multiple Sclerosis: A Randomized Pilot Study
Source: PLoS One. 2016 Jan 22;11(1):e0147195. doi: 10.1371/journal.pone.0147195 (PMC4723316; doi:10.1371/journal.pone.0147195)
Supplement: S1 Table — (DOCX) [file pone.0147195.s006.docx]

| **Antidepressant** | **N** | **Mean dose in mg/day (range)** | **Adequate Minimal Clinical Dose in mg/day** |
| --- | --- | --- | --- |
| Bupropion (wellbutrin) | 2 | 150 (150) | 225 |
| Citalopram (Celexa) | 2 | 20 (20) | 10 |
| Duloxetine (Cymbalta) | 3 | 50 (30-60) | 60 |
| Escitalopram (Lexapro) | 1 | 10 (10) | 10 |
| Fluoxetine (Prozac) | 12 | 34 (10-80) | 10 |
| Paroxetine (Paxil) | 5 | 20 (10-40) | 20 |
| Sertraline (Zoloft) | 7 | 86 (5-200) | 50 |
| Trazodone | 1 | 100 (100) | 150 |
| Venlafaxine (Effexor) | 3 | 138 (113-150) | 75 |

**Supplementary Table 1. Participant Antidepressant Dose**
