# Supplementary material for: Omega-3 Fatty Acids for Depression in Multiple Sclerosis: A Randomized Pilot Study
Source: PLoS One. 2016 Jan 22;11(1):e0147195. doi: 10.1371/journal.pone.0147195 (PMC4723316; doi:10.1371/journal.pone.0147195)
Supplement: S2 Table — (DOCX) [file pone.0147195.s007.docx]

Supplementary Table 2a. Parameter estimates on logistic regression analysis: MADRS 50% improvement from baseline to 3-months

| **Outcome Variable** | **Coefficient for Intervention Group** | **SE (Standard Error)** | **P Value** |
| --- | --- | --- | --- |
| Treatment | 0.89 | 0.85 | 0.30 |
| Age | 0.33 | 0.06 | 0.56 |
| MS disease duration | -0.04 | 0.05 | 0.44 |

Mixed effects logistic regression model adjusted for age and MS disease duration. No difference between placebo and omega-3 FA was found over 3 months (p=0.30).

Supplementary Table 2b. Parameter estimates on linear mixed model analysis: MADRS total score improvement from baseline to 3-months

| **Outcome Variable** | **Coefficient for Intervention Group** | **SE (Standard Error)** | **P Value** |
| --- | --- | --- | --- |
| Treatment | -1.73 | 1.44 | 0.23 |
| Age | -0.11 | 0.09 | 0.25 |
| MS disease duration | 0.08 | 0.09 | 0.37 |

Linear mixed effects model adjusted for age and MS disease duration. No difference between placebo and omega-3 FA was found over 3 months (p=0.23).

Supplementary Table 2c. Parameter estimates on logistic regression analysis: BDI 50% improvement from baseline to 3-months

| **Outcome Variable** | **Coefficient for Intervention Group** | **SE (Standard Error)** | **P Value** |
| --- | --- | --- | --- |
| Treatment | 1.17 | 0.90 | 0.20 |
| Age | -0.10 | 0.08 | 0.18 |
| MS disease duration | 0.02 | 0.05 | 0.72 |

Mixed effects logistic regression model adjusted for age and MS disease duration. No difference between placebo and omega-3 FA was found over 3 months (p=0.20).

Supplementary Table 2d. Parameter estimates on linear mixed model analysis: BDI total score improvement from baseline to 3-months

| **Outcome Variable** | **Coefficient for Intervention Group** | **SE (Standard Error)** | **P Value** |
| --- | --- | --- | --- |
| Treatment | -1.94 | 1.74 | 0.27 |
| Age | 0.02 | 0.11 | 0.85 |
| MS disease duration | 0.03 | 0.10 | 0.74 |

Linear mixed effects model adjusted for age and MS disease duration. No difference between placebo and omega-3 FA was found over 3 months (p=0.27).
